# Whole-genome based strain identification of fowlpox virus directly from cutaneous tissue and propagated virus

**DOI:** 10.1371/journal.pone.0261122

**Published:** 2021-12-16

**Authors:** Kinza Asif, Denise O’Rourke, Alistair R. Legione, Pollob Shil, Marc S. Marenda, Amir H. Noormohammadi

**Affiliations:** 1 Department of Veterinary Biosciences, Asia-Pacific Centre for Animal Health, Faculty of Veterinary and Agricultural Sciences, The University of Melbourne, Werribee, Victoria, Australia; 2 Department of Veterinary Biosciences, Asia-Pacific Centre for Animal Health, Faculty of Veterinary and Agricultural Sciences, The University of Melbourne, Parkville, Victoria, Australia; Ghazi University Dera Ghazi Khan, PAKISTAN

## Abstract

Fowlpox (FP) is an economically important viral disease of commercial poultry. The fowlpox virus (FPV) is primarily characterised by immunoblotting, restriction enzyme analysis in combination with PCR, and/or nucleotide sequencing of amplicons. Whole-genome sequencing (WGS) of FPV directly from clinical specimens prevents the risk of potential genome modifications associated with *in vitro* culturing of the virus. Only one study has sequenced FPV genomes directly from clinical samples using Nanopore sequencing, however, the study didn’t compare the sequences against Illumina sequencing or laboratory propagated sequences. Here, the suitability of WGS for strain identification of FPV directly from cutaneous tissue was evaluated, using a combination of Illumina and Nanopore sequencing technologies. Sequencing results were compared with the sequence obtained from FPV grown in chorioallantoic membranes (CAMs) of chicken embryos. Complete genome sequence of FPV was obtained directly from affected comb tissue using a map to reference approach. FPV sequence from cutaneous tissue was highly similar to that of the virus grown in CAMs with a nucleotide identity of 99.8%. Detailed polymorphism analysis revealed the presence of a highly comparable number of single nucleotide polymorphisms (SNPs) in the two sequences when compared to the reference genome, providing essentially the same strain identification information. Comparative genome analysis of the map to reference consensus sequences from the two genomes revealed that this field isolate had the highest nucleotide identity of 99.5% with an FPV strain from the USA (Fowlpox virus isolate, FWPV-MN00.2, MH709124) and 98.8% identity with the Australian FPV vaccine strain (FWPV-S, MW142017). Sequencing results showed that WGS directly from cutaneous tissues is not only rapid and cost-effective but also provides essentially the same strain identification information as *in-vitro* grown virus, thus circumventing *in vitro* culturing.

## Introduction

FP is a common viral disease of commercial poultry and wild birds caused by avipoxviruses [1]. It is a slow-spreading yet economically important disease, causing a drop in egg production and slow growth of birds followed by mortality [2]. The disease may occur as cutaneous (dry pox) or diphtheritic (wet pox) form [2]. The FPV is characterised by immunoblotting, isolation in CAMs, restriction enzyme analysis in combination with PCR, and/or nucleotide sequencing of amplicons [2, 3]. Polypeptide P4b gene, which is highly conserved among all avipoxviruses is mainly targeted for PCR [4–10]. Based on phylogenetic analysis of the P4b gene, avipoxviruses are divided into three clades and two proposed clades. Clade A includes FP-like viruses, clade B: canary poxviruses, and clade C: parrot poxviruses [11]. Two more proposed clades of avipoxviruses include clade D for quail pox virus and clade E for turkey pox virus [11].

FPV is a large double-stranded DNA virus with a genome of 260–309 kb in size [11, 12]. Currently, next-generation sequencing is being extensively used for the surveillance of poxviruses [13]. Previous studies have determined the complete genome sequences of FPV from the USA [14], China, (Zhao, Y., Liu, S. (2020) [Unpublished]) Germany [15], and Australia [12], using isolation of FPV in chicken embryo fibroblast cells or CAMs, cloning into a plasmid and amplification of PCR product followed by high-throughput sequencing. However, *in vitro* culturing of FPV [1, 16] is not only time-consuming but can also lead to potential genome modifications. *In vitro* isolation of FPVs requires the adaptation of FPV strains when continuous cell lines such as LMH or QT35 are used [2, 17]. Given that the cell culture isolation is less sensitive than CAMs, FPVs are isolated by inoculating in CAMs, however, some strains of FPV may occasionally fail to grow in CAMs and may require adaptation [17]. Only one study has sequenced the FPV genome directly from tracheal and cutaneous tissues using Nanopore sequencing [13]. However, that study did not compare the sequence against a more accurate sequencing technology such as Illumina or the sequence of a laboratory propagated virus. Nanopore sequencing has a higher error rate on average than Illumina sequencing, even though new Nanopore technologies are closing the gap [18].

The complete genome of the vaccine strain of FPV has been sequenced recently in Australia after isolation in chicken embryo skin cells [12], however, there is no complete genome sequence of an Australian FPV field isolate sequenced directly from cutaneous tissue. In this study, we assessed the suitability of next-generation sequencing as a tool for strain identification of FPV directly from the cutaneous tissue using a combination of Illumina and Nanopore sequencing technologies, and to compare the whole genome sequence directly from the tissue with that of the virus grown in CAMs of embryonated chicken eggs.

## Materials and methods

### Case history

The use of embryonated eggs was approved by the Animal Ethics Committee of The University of Melbourne (ethics approval number 1814492). Comb tissue sections used in this study were derived from an individual case submitted to the Asia Pacific Centre for Animal Health in 2018 (accession number W775-18) as part of a disease investigation at an Australian layer flock with multiple sheds. The flock had a history of poxvirus vaccination at 11 weeks of age but had been affected with the cutaneous form of FP at 37 weeks of age with approximately 10% morbidity and slightly lower egg production, swelling of upper eyelids, and nodular lesions on the combs. The cutaneous samples were also tested by FPV PCR [19] and confirmed positive by observing PCR amplicons of the expected size. Small pieces of comb tissue were fixed in neutral formalin, embedded in paraffin, and stained with hematoxylin and eosin. The remaining tissue samples were stored at −80°C until further use for WGS and propagation in CAMs of embryonated chicken eggs.

Pieces of affected comb were grounded with sterile mortar and pestle followed by resuspension in Medium 199 (Gibco™) containing 2 mM L-glutamine, 50 μg/mL of penicillin, 50 μg/mL of streptomycin, and 5 μg/mL of amphotericin B and kept at room temperature for 1 hour. After 1 hour, the suspension was centrifuged at 3900 × *g* for 10 min at 4°C and the supernatant was used for virus isolation or DNA extraction.

### Virus isolation and propagation

For virus inoculation, 100 μL of 1:10 diluted supernatant was inoculated into CAMs of 10-day-old specific pathogen-free chicken embryos. Inoculated eggs were incubated at 37°C for 5 days, followed by the collection of CAMs containing pocks. The collected CAMs were homogenised and kept at -80°C until used for DNA extraction.

### Viral DNA extraction

The homogenised CAMs were frozen and thawed three times and centrifuged at 3900 × *g* for 30 min at 4°C. Supernatants obtained directly from the comb and CAMs were processed for DNA extraction using ultracentrifugation as described previously [20]. Briefly, the supernatant was layered onto a 30% w/v ice-cold sucrose cushion in an open-top tube, and virions were pelleted at 98,000 × *g* for 90 min at 4°C. The supernatant was removed, and pellet was resuspended in 2 × lysis buffer (0.01 M Tris-HCl [pH 7.5], 0.01 M EDTA [pH 8.0], 1% SDS, 1 mg/mL proteinase K in 0.01 M Tris-HCl [pH 8]) and incubated overnight at 37°C. Samples directly from comb and CAMs were named FPV-COMB and FPV-CAMs respectively.

The DNA from FPV-COMB and FPV-CAMs was extracted using phenol-chloroform as described previously [20]. Briefly, one volume of buffered phenol, chloroform, and isoamyl alcohol (ratio 25:24:1, respectively) [21] was added to the lysed virus and the mixture centrifuged at 18,000 × *g* for 10 min at 4°C. The aqueous phase was transferred into a new centrifuge tube and treated as above but using chloroform and isoamyl alcohol only (ratio 24:1 respectively). DNA was precipitated using 1/10 volume of 3 M sodium acetate and 1 volume of ice-cold absolute ethanol followed by centrifugation at 18,000 × *g* for 20 min at 4°C and washing with 70% ethanol. The resultant pellets were air-dried and dissolved in 100 μL of nuclease-free water. The quantity and quality of DNA were measured using Qubit fluorometer (Invitrogen) and NanoDrop™ 1000 spectrophotometer (Thermo Fisher Scientific). Viral DNA extracted from comb and CAMs was stored at -20°C until used for PCR and genome sequencing.

### Nanopore sequencing and data analysis

Oxford Nanopore sequencing libraries of FPV-COMB and FPV-CAMs were prepared using SQK-LSK108 and SQK-LSK308 kits respectively as per the manufacturer’s instructions. Sequencing was allowed to run for 24 hours on a MinION MK-1b device fitted with a FLO-MIN107 (R9.5 chemistry) flow cell. Raw 1D data in fast5 format was base called using guppy_basecaller with basecall model of high accuracy (version 4.0.15) (Oxford Nanopore Technologies) using the University of Melbourne High-Performance Cluster, Spartan [22]. Porechop (version 0.2.3) (https://github.com/rrwick/Porechop) was used to trim the adapter sequences. FASTQ WIMP (version 2021.03.05) analysis was performed using the EPI2ME Desktop agent (version 3.3.0) (Oxford Nanopore Technologies) to investigate the taxonomic diversity of both isolates (FPV-COMB and FPV-CAMs). Nanopore reads with a minimum length of 1000 bp were extracted from a set of FASTQ files using Filtlong (version 0.2.0) (https://github.com/rrwick/Filtlong). Trimmed sequence reads were mapped against the *Gallus gallus* reference genome (GenBank NC_006088) using Minimap2 (2.17-r941) [23] to remove chicken genome-associated reads from the data. Unmapped reads from this step were mapped against a *Staphylococcus aureus* reference genome (GenBank AP017922.1) to remove the major bacterial taxa sequenced.

### Illumina sequencing and data analysis

Illumina sequencing of FPV-COMB isolate was performed at Deakin Genomics Centre, Burwood, Victoria, Australia. Sequencing libraries were prepared with the Illumina DNA prep kit and sequenced on the Illumina MiSeq 2 x 300bp V3 chemistry whilst FPV-CAMs Illumina sequencing was performed at Walter and Eliza Hall Institute of Medical Research, Melbourne, Australia using the Illumina NextSeq 300 platform, and libraries were prepared according to the TruSeq DNA protocol. The computational analysis utilised the Galaxy Europe workflow manager (https://usegalaxy.eu/). For pre-processing of Illumina reads, Fastp (Galaxy version 0.20.1) [24] was used for detection and removal of adapters and bases below a Phred quality score of 20. For taxonomic classification of both FPV-COMB and FPV-CAMs, Kraken2 (Galaxy version 2.1.1+galaxy1) [25] database “viral genome 2019” was used and results were visualised with Krona Pie chart (Galaxy Version 2.6.1) [26]. The quality check of paired Illumina reads was performed using FastQC [27]. Trimmed Illumina reads were mapped against *Gallus gallus* and *Staphylococcus aureus* as mentioned earlier for Nanopore reads.

Unmapped Nanopore and Illumina reads were used as input data for hybrid assembly using Unicycler (Galaxy version 0.4.8.0) [28] for both FPV-COMB and FPV-CAMs. Nanopore and Illumina non-hybrid *de novo* assemblies were generated using Canu (version 1.7) [29] and Unicycler, respectively. Contigs were assessed by BLAST (Blast+ version 2.11.0) against the entire NBCI nucleotide (nt) database on the University of Melbourne High-Performance Cluster. This process used the megablast algorithm and a minimum E-value of 10^−5^ for high confidence hits. Each genome was annotated with genome annotation utility transfer (GATU) [30] using FWPV-MN00.2 (MH709124) as the reference.

### Comparative genome analyses

Trimmed Illumina reads from FPV-COMB and FPV-CAMs were mapped to the FPV reference genome (FWPV-MN00.2, MH709124) using Bowtie2 (version 2.3.0) [31] plugin in Geneious Prime^®^ (version 2021.1.1) [32] to generate map to reference consensus sequences. Nucleotide sequence alignments were conducted with MAFFT [33], using the method FFT-NS-I in Geneious Prime^®^ using reference genomes and map to reference consensus sequences of FPV-COMB and FPV-CAMs. Screening for SNPs was conducted using Snippy (version 4.6.0+galaxy0) [34] using the FPV genome (FWPV-MN00.2, MH709124) as reference. To investigate the presence and length of integrated reticuloendotheliosis provirus (REV), map to reference consensus sequences were separately aligned to REV strain 104865 (KJ756349). The evolutionary relationship of Australian FPV-COMB and FPV-CAMs was determined by constructing a phylogenetic tree using the neighbour-joining method, a Kimura 2-parameter model with 1000 bootstrap iterations in Mega 7 (version 7.0.26) [35] based on complete genome sequences.

## Results

### Histopathological observations

**Fig 1:**
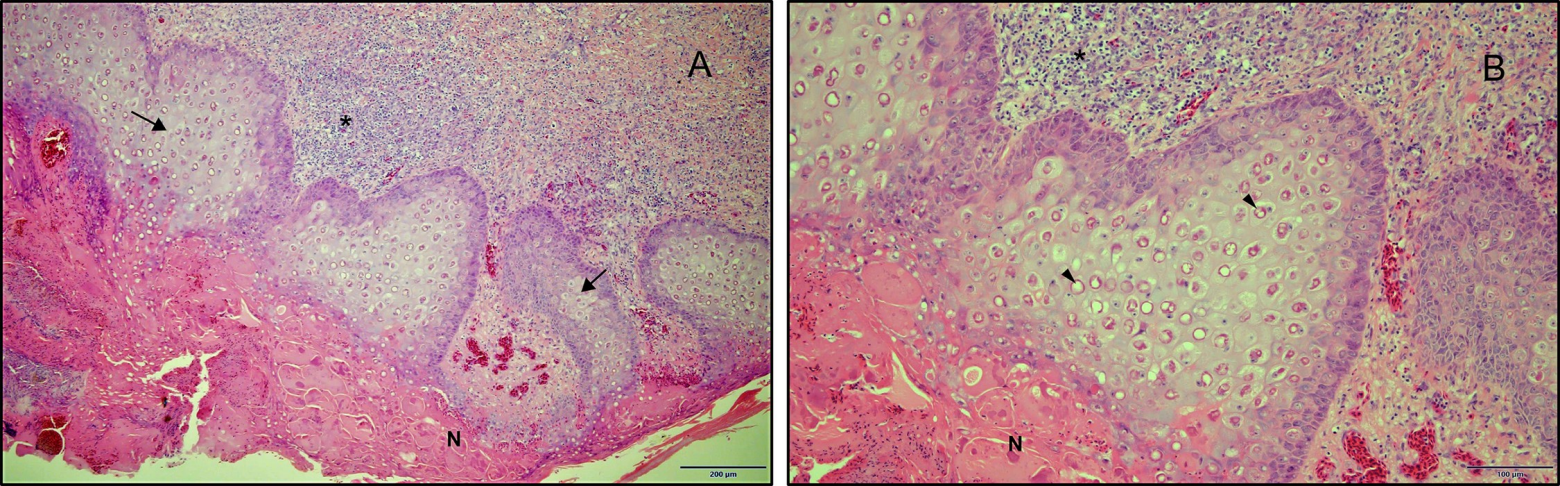
Low (A) and high (B) magnification histopathological images of FPV field isolate from comb tissue showing erosion of the epithelial lining in association with necrotic cell debris (N) oedema and mononuclear inflammatory cell infiltration into the dermis (*), hyperplasia and ballooning of the epithelial cells (arrows in A) and round intracytoplasmic eosinophilic inclusion bodies in some of the epithelial cells (arrowheads in B).

Histopathological examination of several cross-sections of comb tissues showed lesions consistent with FP including erosion of the epithelial lining in association with necrotic cell debris, subcutaneous oedema and mononuclear inflammatory cell infiltration into dermis; hyperplasia and ballooning of the epithelial cells; and the presence of round intracytoplasmic eosinophilic inclusion bodies in some epithelial cells (Fig 1A and 1B). Also, in some places, metaplasia of the epithelial lining and large number of bacterial colonies (cocci) in amongst the superficial necrotic cell debris were detected (not shown in the images).

### Sequencing analysis and comparison

Details of the sequencing reads obtained from Nanopore, and Illumina sequencing platforms of both FPV-CAMs and FPV-COMB are summarised in Table 1.

**Table 1:** Details of the sequencing reads before and after quality filtering and assembly.

	FPV-CAMs		FPV-COMB	
	Nanopore	Illumina (P.E)	Nanopore	Illumina (P.E)
Total reads	268,810	2,130,746	152,549	254,555
Filtered reads	70,670	1,843,348	10,049	234,323
Mean read length	2081	151	1523	300
Maximum read length	27,007	151	47,327	300
Read N50	2216	--	1466	--
Total bases	147,123,820	546,125,392	15,311,721	113,154,919
Reads mapped to *Gallus gallus*	16,989/53,681^a^	456,247/1,387,100^a^	2436/7613^a^	59,931/174,392^a^
Reads mapped to *Staph*. *aureus*	227/53,454^a^	5513/1,381,587^a^	13/7600^a^	1101/173,291^a^
*De novo* assembly (bp)	Incomplete	278,519	Could not assemble	298,870
Hybrid assembly size (bp)^b^	278,519		298,906	

^a^ Mapped/unmapped reads.

^b^ Hybrid assembly generated from Unicycler.

#### Sequencing analysis of FPV-CAMs

**Fig 2:**
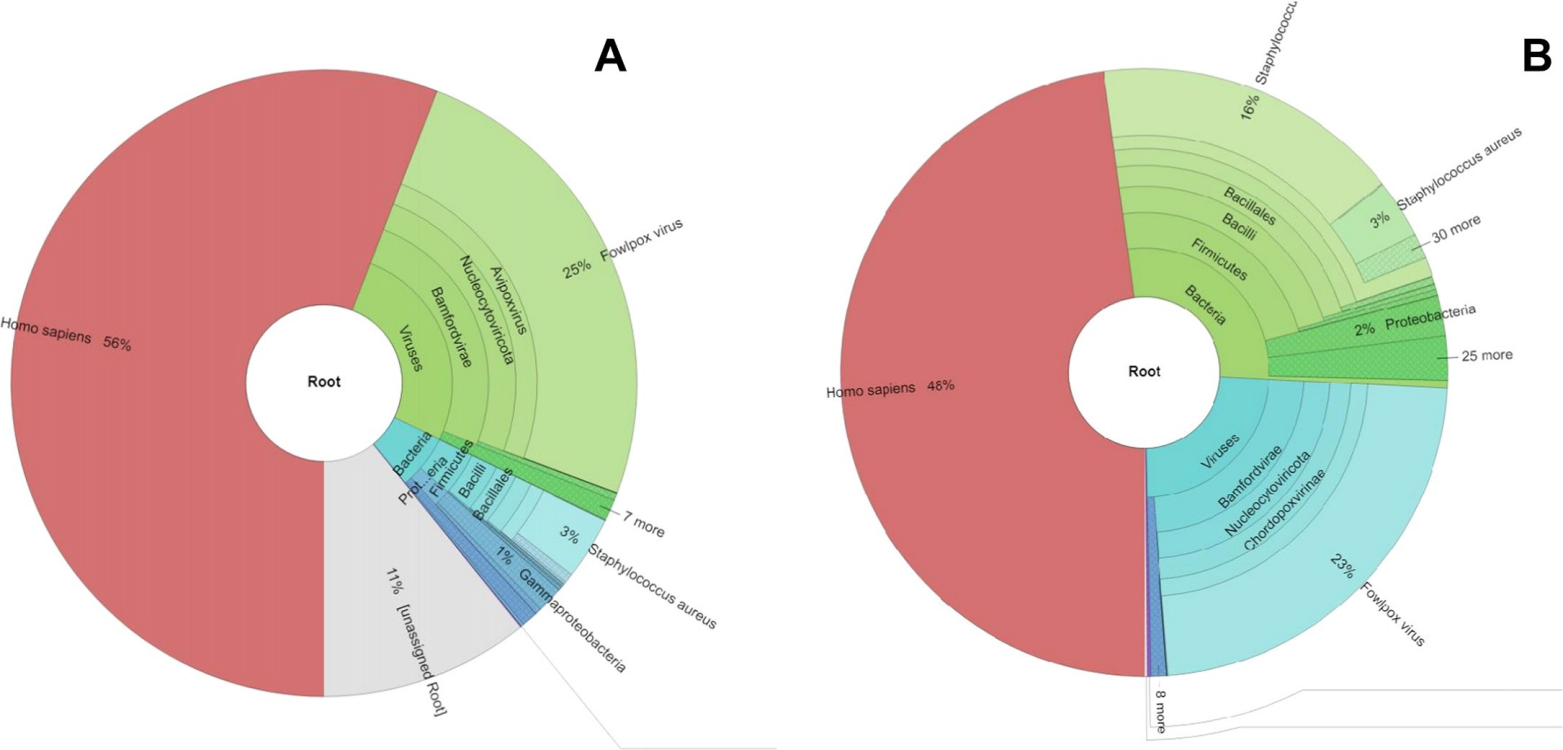
Taxonomic classification of FPV-CAMs (A) and FPV-COMB (B) using Kraken2 and visualised by Krona pie chart. Reads identified as homo sapiens are a result of *Gallus gallus* not being present in the smaller Kraken2 database utilised.

Briefly, a total of 2,130,746 paired-end Illumina short reads and 268,810 Nanopore long reads were generated for FPV-CAMs. After trimming, 1,843,348 Illumina reads and 70,670 Nanopore reads were obtained. Taxonomic classification of the Nanopore reads from FPV-CAMs by WIMP, considering organisms with more than 100 hits, was as follows: 67%, eukaryotic; 5%, bacterial (predominantly genus *Staphylococcus*); and 28%, viral (including genus *Avipoxvirus* and *Gammaretrovirus*). Taxonomic classification of Illumina reads from FPV-CAMs by Kraken2 database “viral genomes 2019” classified 9.5% (204,039) of the reads, with the remaining unclassified reads identified at >95% hits with *Gallus gallus* using the Centrifuge classification (version 1.0.3) at the University of Melbourne High-Performance Cluster, Spartan. Taxonomic classification of classified reads from Kraken2 showed 56% of the reads classified as homo sapiens, 7% bacterial (predominantly genus *Staphylococcus*), and 25% viral (genus *Avipoxvirus* and *Gammaretrovirus*) (Fig 2A). Mapping of trimmed reads against *Gallus gallus* and *Staphylococcus aureus* resulted in a total of 1,381,587 unmapped Illumina short reads and 53,454 Nanopore long reads. Using unmapped reads (from the previous step) as input for hybrid assembly, 30 contigs were generated with the largest being 278,519 bp. Analysis with BLAST revealed 1/30 contigs with a best-hit matching to FPV (S1 Table). *De novo* assembly of Illumina reads generated 63 contigs with the largest being 278,519 bp, and 1/63 contigs with BLAST hits to FPV genome sequences (S2 Table). A single contig from both Illumina and hybrid *de novo* assemblies contained a nearly complete genome of FPV excluding right and left terminal repeat regions. Nanopore *de novo* assembly generated 8 contigs with the largest being 56,383 bp and 7/8 contigs with BLAST best hits to FPV genome sequences (S3 Table). Iterative mapping of 7 selected contigs resulted in the genome of 270,964 bp with four major gaps ranging in sizes from 600 bp to 16,000 bp in the genome. The remaining contigs belonged to the *Gallus gallus* genome based on BLAST analysis.

#### Sequencing analysis of FPV-COMB

A total of 254,555 paired-end Illumina short reads and 152,549 Nanopore long reads were generated for FPV-COMB. After filtering, a total of 234,323 Illumina reads and 10,049 Nanopore reads were utilised. Taxonomic classification of the Nanopore reads from FPV-COMB by WIMP was as follows: 75%, eukaryotic; 18%, bacterial (predominantly genus *Staphylococcus*); and 7% viral (including genus *Avipoxvirus* and *Gammaretrovirus*). Taxonomic classification of Illumina reads from FPV-COMB by Kraken2 classified 12.6% (31,956) of the reads classified and the remaining unclassified reads were identified as *Gallus gallus*. From classified reads, Kraken2 resulted in 48% reads as eukaryotic, 16% bacterial (predominantly genus *Staphylococcus*), and 23% viral (genus *Avipoxvirus* and *Gammaretrovirus*) (Fig 2B). Mapping of trimmed reads against *Gallus gallus* and *Staphylococcus aureus* produced a total of 173,291 unmapped Illumina reads and 7600 Nanopore long reads for assembly. The resultant hybrid assembly from unmapped reads generated 77 contigs with the largest being 149,786 bp. BLAST analysis of contigs revealed that 4/77 contigs with BLAST hit to FPV genome sequences (S4 Table). *De novo* assembly of Illumina reads generated 118 contigs with the largest being 157,491 bp, and 4/118 contigs with BLAST hits to FPV (S5 Table). Iterative mapping of selected 4 contigs for Illumina and hybrid *de novo* assemblies resulted in complete genomes of 298,870 and 298,906 bp respectively. The remaining contigs were identified as being from *Gallus gallus* based on BLAST hits. Nanopore *de novo* assembly generated only 3 contigs with the largest contig of 19,283 bp and none of these contigs were from FPV according to BLAST (S6 Table).

### Comparison of the map to reference consensus sequences using Illumina and Nanopore reads

Mapping of FPV-CAMs filtered Illumina reads from the previous step against FPV reference genome (FWPV-MN00.2, MH709124) mapped 104,357 reads with the mean coverage of 48 and a map to reference consensus sequence of 298,739 bp was obtained. Mapping of Nanopore reads of FPV-CAMs against the reference genome mapped 7173 reads with the mean coverage of 27 and a resultant sequence of 299,153 bp.

For FPV-COMB, a total of 14,836 Illumina reads mapped to the reference with the mean coverage of 13 and resulted in a map to reference consensus genome of 298,250 bp. Mapping of Nanopore reads of FPV-COMB against the reference genome mapped 151 reads with the mean coverage of 0.8 and an incomplete genome sequence with multiple gaps throughout the genome. Therefore, map to reference consensus sequences of FPV-COMB and FPV-CAMs obtained after mapping Illumina reads were used for further comparative genome and phylogenetic analyses unless stated otherwise.

A total of 259 coding sequences (CDS) were annotated for both FPV-COMB and FPV-CAMs map to reference consensus sequences. The GC content was 31.4% in both FPV-COMB and FPV-CAMs.

Map to reference consensus sequences of both FPV-CAMs and FPV-COMB were submitted in GenBank under accession numbers OK558608 and OK558609 respectively.

### Polymorphism analysis indicates the presence of a nearly identical number of SNPs in FPV-COMB and FPV-CAMs

Polymorphism analysis using Snippy indicated the presence of 204 SNPs in FPV-CAMs and 152 in FPV-COMB when compared to the reference genome (FWPV-MN00.2, MH709124). Comparison of SNPs between FPV-CAMs and FPV-COMB revealed that the 152 SNPs in FPV-COMB also existed exactly at identical positions to that of FPV-CAMs while 52 SNPs were not found in FPV-COMB. Upon manual inspection of the map to reference results these 52 SNPs were reflected in the reads obtained from FPV-COMB, however, the depth of coverage at these positions fell below the minimum coverage of 10 utilised by Snippy to identify SNPs with confidence.

### Map to reference consensus sequences of Australian FPV-COMB and FPV-CAMs carry near full-length reticuloendotheliosis provirus

**Fig 3:**
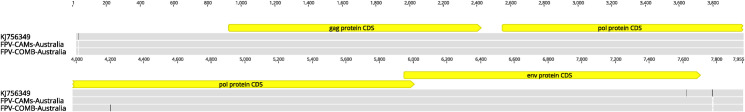
Comparison of near-full length provirus of REV of Australian FPV-COMB and FPV-CAMs with REV strain 104865 (KJ756349).

A large 7932 bp fragment containing a near full-length genome of REV containing all three genes (gag, env, pol) was found in the map to reference consensus sequences of FPV-CAMs and FPV-COMB. The REV integration occurred between nucleotide positions 233,105–241,036 and 232,879–240,810 of the genome in FPV-CAMs and FPV-COMB respectively. BLAST searches revealed that integrated full-length REV had 99.9% nucleotide identity to REV strain 104865 (KJ756349) (Fig 3).

### Australian FPV field isolate showed the highest nucleotide identity to the FPV strain from the USA

**Fig 4:**
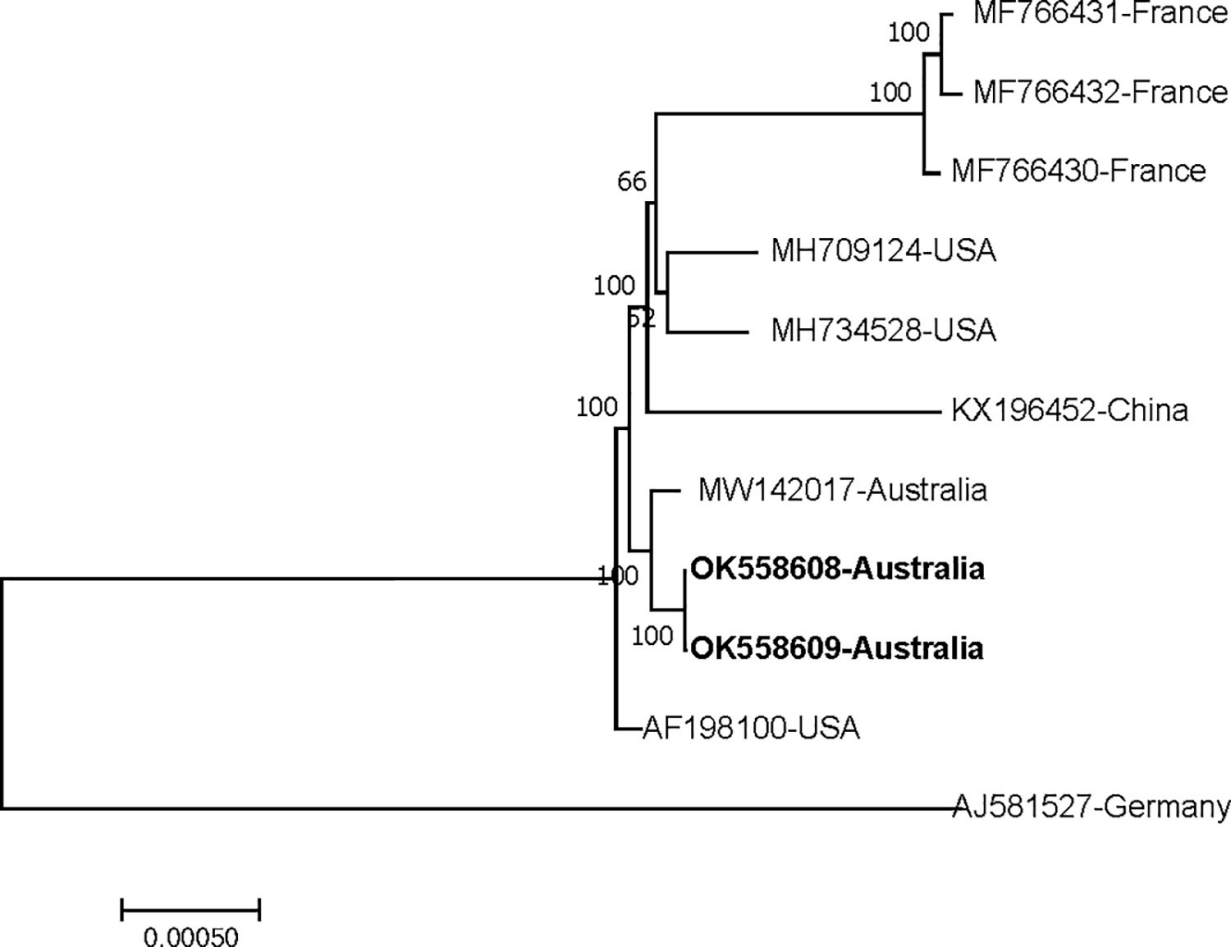
Genetic relationship of Australian FPV-COMB and FPV-CAMs with reference sequences selected from previous studies. The relationship was inferred based on phylogenetic analysis of complete genome sequences using the neighbour-joining method with 1000 bootstrap iterations. The labels at branch tips refer to the GenBank accession number followed by the name of the country. Australian FPV-COMB (OK558609) and FPV-CAMs (OK558608) are highlighted in bold.

Nucleotide sequence alignments with previously sequenced FPV strains revealed that Australian FPV-CAMs and FPV-COMB showed the highest nucleotide identity of 99.5% to the FPV strain from the USA (FWPV-MN00.2, MH709124) followed by 98.8% with the FPV vaccine strain from Australia (MW142017). The homology comparison of complete genomes of Australian FPV-CAMs and FPV-COMB along with the reference genomes is given in Table 2. Subsequent phylogenetic analysis positioned Australian FPV-COMB and FPV-CAMs, closer to Australian FPV vaccine strain (FWPV-S, MW142017), in a clade with other FPVs from the USA (bootstrap support, 100%) (Fig 4).

**Table 2:** The homology comparison of complete genomes of Australian FPV-COMB and FPV-CAMs with previously published reference genomes.

	MH709124-USA	(OK558608)-Australia	(OK558609)—Australia	MW142017-Australia	MH734528-USA	AF198100-USA	KX196452-China	MF766430-France	MF766431-France	MF766432-France	AJ581527-Germany
**MH709124**		99.6	99.5	98.6	99.0	96.5	94.5	96.2	96.4	96.4	87.8
**FPV-CAMs**	99.6		99.8	98.7	99.0	96.6	94.5	96.2	96.5	96.5	87.8
**FPV-COMB**	99.5	99.8		98.8	99.2	96.7	94.7	96.2	96.5	96.5	87.8
**MW142017**	98.6	98.7	98.8		99.4	95.9	94.7	95.8	95.7	95.7	86.9
**MH734528**	99.0	99.0	99.2	99.4		96.1	94.8	96.1	96.0	96.0	87.2
**AF198100**	96.5	96.6	96.7	95.9	96.1		96.7	99.3	99.6	96.6	90.5
**KX196452**	94.5	94.5	94.7	94.7	94.8	96.7		96.4	96.6	96.6	91.4
**MF766430**	96.2	96.2	96.2	95.8	96.1	99.3	96.4		99.5	99.5	90.2
**MF766431**	96.4	96.5	96.5	95.7	96.0	99.6	96.6	99.5		99.9	90.4
**MF766432**	96.4	96.5	96.5	95.7	96.0	99.6	96.6	99.5	99.9		90.4
**AJ581527**	87.8	87.8	87.8	86.9	87.2	90.5	91.4	90.2	90.4	90.4	

## Discussion

In this study, WGS of Australian FPV field isolate was attempted using viral DNA extracted directly from cutaneous tissue and virus-infected CAMs. Theoretically, WGS of a pathogen directly from clinical samples prevents the risk of genome modifications that could occur after passage *in vitro* [36–38]. The additional challenge with poxvirus is that it is difficult to propagate in cell culture and cultured genomes might not represent the true picture of *in vivo* infection, therefore WGS directly from clinical samples remains the most suitable option for characterisation of large viral genomes such as poxvirus [37]. Here, the suitability of WGS as a tool for strain identification of FPV directly from cutaneous tissues was evaluated. Given that clinical samples for FP are only infrequently submitted, and of all the samples submitted, this individual sample was selected for this study because this was confirmed positive by PCR and histopathology. Data obtained from cutaneous tissue using Illumina sequencing generated reads of high-quality and coverage and the resultant map to reference consensus sequence generated a complete genome of 298,250 bp. In contrast, due to the smaller number of Nanopore reads for FPV-COMB, mapping was compromised by poor overlapping and gaps, and generating a map to reference consensus sequence was not possible. It is noteworthy that the specimen used for WGS have contained very small nodular lesions that may have contributed to the small number of reads generated by the Nanopore sequencing platform. Nevertheless, based on these results obtained for FPV-COMB, Nanopore sequencing would be useful to determine the presence of FPV using kmer matching methods such as Kraken2 or long-read map to reference methods such as Minimap2. However, for in-depth genomic information such as strain identification, Illumina sequencing directly from cutaneous tissues is a suitable option. Data obtained from the grown virus (FPV-CAMs) generated high-quality reads using both Nanopore and Illumina sequencing and map to reference consensus sequences resulted in complete genomes of ~298 kb. Comparison of the map to reference consensus sequences of both FPV-COMB and FPV-CAMs revealed nucleotide identity of 99.8% between the two. This confirms the ability of WGS directly from cutaneous tissues to accurately genotype FPV. It is therefore tempting to conclude that genomic sequencing directly from clinical tissues is not only fast, but also generates a complete genome sequence similar to a grown FPV. Moreover, sequencing directly from cutaneous tissues reduces the cost by not requiring fertilised embryonated eggs nor the requisite incubation equipment. There are dramatic potential improvements in time-based efficiency using sequencing approaches as well, with isolation of FPV in embryonated eggs taking 2–3 weeks for the embryo to reach the appropriate stage for inoculation, the growth of the virus, and subsequent typing. Moreover, a comparison of SNPs revealed the presence of an identical number of SNPs in FPV-COMB similar to FPV-CAMs, providing the same typing information as a grown virus. It is noteworthy that both FPV-COMB and FPV-CAMs sequences are from the same virus and thus a comparable number of SNPs is expected unless *in vitro* mutations have occurred. In this study, optimal results for comparative genome analysis were obtained with sufficient depth using a map to reference approach with Illumina reads. Given that the FPV genome is relatively syntenic, the map to reference approach is suitable for rapid characterisation of the FPV genome.

Genome comparison with previously sequenced FPVs revealed that the Australian FPV-COMB and FPV-CAMs showed the highest nucleotide identity of 99.5% to FPV strain from the USA (FWPV-MN00.2, MH709124) and 98.8% to the Australian FPV vaccine strain (FWPV-S, MW142017). Based on phylogenetic analysis of complete genomes, Australian FPV-COMB and FPV-CAMs were positioned closer to the Australian FPV vaccine strain, in a clade with other FPVs from the USA with 100% bootstrap support.

Most FPV field isolates carry integrated REV which indicates natural genetic engineering in viruses [39]. Here, the presence of a near full-length genome (7932 bp) of REV provirus was reported in both FPV-COMB and FPV-CAMs based on WGS. Previously, the presence of near full-length REV provirus has been reported in vaccine and field isolates of FPV circulating in Australia based on hybridization techniques, PCR, and sequencing [12, 40]. Although the role of REV is unclear, they have been associated with high virulence of FPV (1) and immunosuppressive effects in young chickens [39].

From comparative genome analyses, it can be concluded that the WGS directly from cutaneous tissues is rapid, cost-effective, and provides essentially the same strain identification information as obtained from the FPV grown in CAMs. The scarcity of fowlpox virus full genomes highlights the importance of our research outlining a new way to obtain this data and this work will lead to an expansion of the availability of complete genomes covering a wider geographical area. Considering the syntenic genome of FPV, it is proposed that reference-guided sequencing analysis could be a better option for sequencing directly from clinical specimens. Future studies should include the screening of a large number of specimens from different strains of birds and different geographical locations to put this hypothesis to test. Also, it would be interesting to know if diphtheritic lesions found in the wet form of avian pox can potentially generate comparable WGS reads to that of the cutaneous lesions reported here.

## Supporting information

S1 Table Analysis of FPV-CAMs hybrid assembly contigs with BLAST. (DOCX)

S2 Table Analysis of FPV-CAMs Illumina *de novo* assembly contigs with BLAST. (DOCX)

S3 Table Analysis of FPV-CAMs Nanopore *de novo* assembly contigs with BLAST. (DOCX)

S4 Table Analysis of FPV-COMB hybrid assembly contigs with BLAST. (DOCX)

S5 Table Analysis of FPV-COMB Illumina *de novo* assembly contigs with BLAST. (DOCX)

S6 Table Analysis of FPV-COMB Nanopore *de novo* assembly contigs with BLAST. (DOCX)
