# Supplementary material for: Whole-genome based strain identification of fowlpox virus directly from cutaneous tissue and propagated virus
Source: PLoS One. 2021 Dec 16;16(12):e0261122. doi: 10.1371/journal.pone.0261122 (PMC8675702; doi:10.1371/journal.pone.0261122)
Supplement: S1 Table — (DOCX) [file pone.0261122.s001.docx]

**S1 Table. Analysis of FPV-CAMs hybrid assembly contigs with BLAST.**

| Contigs | % Identity | Alignment length | Subject Accession^a^ | Subject title^b^ |
| --- | --- | --- | --- | --- |
| 1 | 99.954 | 187932 | AF198100 | Fowlpox virus, complete genome |
| 2 | 99.67 | 11504 | MH879470 | Gallus gallus mitochondrion, |
| 3 | 98.162 | 10391 | AC275676 | gallus gallus BAC clone J_AA005I07 |
| 4 | 97.402 | 6889 | AC191954 | Gallus gallus BAC clone TAM33- |
| 5 | 99.923 | 1305 | XR_003071330 | PREDICTED: Gallus gallus borealin-like (LOC107057188) |
| 6 | 89.869 | 3425 | MG967540 | Gallus gallus 5' external transcribed spacer 18S ribosomal RNA gene |
| 7 | 99.354 | 1703 | XR_003071693 | Gallus gallus uncharacterized |
| 8 | 99.496 | 595 | AB556513 | Gallus gallus DNA |
| 9 | 99.698 | 1985 | XM_025145577 | PREDICTED: Gallus gallus |
| 10 | 87.279 | 621 | AB556728 | Gallus gallus DNA, chromosome 11 |
| 11 | 99.169 | 1684 | XM_025146527 | Gallus gallus coiled-coil domain-containing protein 81-like (LOC107057251) |
| 12 | 99.759 | 2493 | AB556728 | Gallus gallus DNA, chromosome 11 |
| 13 | 98.274 | 3418 | AC204734 | Gallus gallus BAC clone CH261-78A20 |
| 14 | 99.838 | 2475 | MG967540 | Gallus gallus 5' external transcribed spacer 18S ribosomal RNA gene |
| 15 | 97.846 | 2089 | AB556723 | Gallus gallus DNA, chromosome 2 |
| 16 | 99.439 | 1070 | X57344 | G.gallus repetitive DNA |
| 17 | 99.856 | 2781 | AC215792 | Gallus gallus BAC clone CH261- |
| 18 | 90.642 | 1090 | XM_015290599 | Gallus gallus uncharacterized |
| 19 | 91.649 | 1413 | XR_003077428 | Gallus gallus uncharacterized |
| 20 | 99.92 | 2505 | AC232991 | Gallus gallus FOSMID clone J_AD-606H7 |
| 21 | 99.861 | 1438 | AB556724 | Gallus gallus DNA, chromosome 3 |
| 22 | 83.824 | 204 | AC189679 | Gallus gallus BAC clone CH261-81E3 |
| 23 | 98.723 | 1880 | AB556726 | Gallus gallus DNA, chromosome 7 |
| 24 | 99.887 | 1774 | AB556734 | Gallus gallus DNA, chromosome 1 |
| 25 | 79.71 | 1242 | MK388403 | Staphylococcus aureus strain pa2 plasmid pAvY-B1 |
| 26 | 98.944 | 947 | AF124927 | Gallus gallus clone pG6416 inverted repeat region |
| 27 | 99.93 | 1428 | XR_003073179 | Gallus gallus uncharacterized |
| 28 | 83.564 | 578 | XM_015288023 | Gallus gallus translation initiation factor IF-2-like |
| 29 | 87.063 | 943 | X57344 | G.gallus repetitive DNA |
| 30 | 90.994 | 533 | M24755 | Gallus gallus clone pUGD0601 |

^a^Accession number of the best match genome

^b^Name of the best match genome
