# Supplementary material for: Whole-genome based strain identification of fowlpox virus directly from cutaneous tissue and propagated virus
Source: PLoS One. 2021 Dec 16;16(12):e0261122. doi: 10.1371/journal.pone.0261122 (PMC8675702; doi:10.1371/journal.pone.0261122)
Supplement: S2 Table — (DOCX) [file pone.0261122.s002.docx]

**S2 Table. Analysis of FPV-CAMs Illumina *de novo* assembly contigs with BLAST.**

| Contigs | % Identity | Alignment length | Subject Accession^a^ | Subject title^b^ |
| --- | --- | --- | --- | --- |
| 1 | 99.954 | 187932 | AF198100 | Fowlpox virus, complete genome |
| 2 | 99.67 | 11504 | MH879470 | Gallus gallus mitochondrion |
| 3 | 97.881 | 10809 | AC275676 | gallus gallus BAC clone J_AA005I07 |
| 4 | 98.357 | 9800 | AC233985 | Gallus gallus BAC clone CH261-168N7 |
| 5 | 98.797 | 2162 | XR_003071678 | Gallus gallus uncharacterized |
| 6 | 99.846 | 3895 | MG967540 | Gallus gallus 5' external transcribed spacer 18S ribosomal RNA gene |
| 7 | 99.923 | 1305 | XR_003071330 | Gallus gallus borealin-like (LOC107057188) |
| 8 | 99.496 | 595 | AB556513 | Gallus gallus DNA, CENP-A associated sequence |
| 9 | 97.737 | 2607 | AC275676 | gallus gallus BAC clone J_AA005I07 |
| 10 | 99.332 | 5842 | AC270364 | Gallus gallus fosmid J_AD-67N6 |
| 11 | 99.662 | 5624 | AC270329 | Gallus gallus fosmid J_AE-33C7 |
| 12 | 96.368 | 4571 | AC191954 | Gallus gallus BAC clone TAM33-44P13 |
| 13 | 98.071 | 3836 | AC186352 | Gallus gallus BAC clone CH261-143E22 |
| 14 | 98.579 | 3731 | AC232992 | Gallus gallus FOSMID clone J_AD-669E23 |
| 15 | 99.839 | 1240 | XR_003073353 | Gallus gallus uncharacterized |
| 16 | 98.383 | 3401 | AC188439 | Gallus gallus BAC clone CH261-21D19 |
| 17 | 99.729 | 2214 | AB556728 | Gallus gallus DNA, chromosome 11 |
| 18 | 99.423 | 1733 | AB556723 | Gallus gallus DNA, chromosome 2 |
| 19 | 97.882 | 2927 | AC190410 | Gallus gallus BAC clone TAM32-18E12 |
| 20 | 99.856 | 2781 | AC215792 | Gallus gallus BAC clone CH261-98P16 |
| 21 | 90.642 | 1090 | XM_015290599 | Gallus gallus uncharacterized |
| 22 | 91.366 | 1413 | XR_003077428 | Gallus gallus uncharacterized |
| 23 | 96.484 | 2531 | AC191646 | Gallus gallus BAC clone CH261-163E17 |
| 24 | 99.96 | 2505 | AC232991 | Gallus gallus FOSMID clone J_AD-606H7 |
| 25 | 97.882 | 2502 | AC192750 | Gallus gallus BAC clone CH261-164E9 |
| 26 | 83.824 | 204 | AC189679 | Gallus gallus BAC clone CH261-81E3 |
| 27 | 99.549 | 1108 | XR_003073179 | PREDICTED: Gallus gallus uncharacterized |
| 28 | 87.842 | 732 | AB556728 | Gallus gallus DNA, chromosome 11 |
| 29 | 99.645 | 1408 | AB556724 | Gallus gallus DNA, chromosome 3 |
| 30 | 97.917 | 1920 | AC204441 | Gallus gallus BAC clone CH261-160P4 |
| 31 | 99.906 | 1066 | AB556726 | Gallus gallus DNA, chromosome 7 |
| 32 | 99.676 | 1854 | AC215792 | Gallus gallus BAC clone CH261-98P16 |
| 33 | 100 | 314 | XM_025145394 | PREDICTED: Gallus gallus olfactory receptor 14J1-like |
| 34 | 98.82 | 1779 | AC208392 | Gallus gallus BAC clone TAM32-4J23 |
| 35 | 99.887 | 1774 | AB556734 | Gallus gallus DNA, chromosome 1 |
| 36 | 88.732 | 71 | AC270447 | Gallus gallus fosmid J_AD-49G16 |
| 37 | 98.804 | 1672 | AC216221 | Gallus gallus BAC clone CH261-8M6 |
| 38 | 98.516 | 1550 | AC200646 | Gallus gallus BAC clone CH261-126P15 |
| 39 | 89.507 | 629 | X57344 | G.gallus repetitive DNA |
| 40 | 88.264 | 622 | AB556668 | Gallus gallus DNA, CENP-A associated sequence |
| 41 | 79.71 | 1242 | MK388403 | Staphylococcus aureus strain pa2 plasmid pAvY-B1 |
| 42 | 97.368 | 1368 | AY491003 | Gallus gallus clone PG6417 |
| 43 | 99.781 | 1371 | KT445934 | Gallus gallus 5' external transcribed spacer, 18S ribosomal RNA gene |
| 44 | 96.183 | 786 | CR390638 | Gallus gallus finished cDNA |
| 45 | 89.731 | 633 | AB556456 | Gallus gallus DNA, CENP-A associated sequence |
| 46 | 74.839 | 620 | AB556456 | Gallus gallus DNA, CENP-A associated sequence |
| 47 | 87.5 | 360 | AB556671 | Gallus gallus DNA, CENP-A associated sequence |
| 48 | 83.564 | 578 | XM_015288023 | PREDICTED: Gallus gallus translation initiation factor IF-2-like |
| 49 | 90.099 | 505 | X57344 | G.gallus repetitive DNA |
| 50 | 98.229 | 621 | AB556668 | Gallus gallus DNA, CENP-A associated sequence |
| 51 | 99.295 | 1134 | AC275676 | gallus gallus BAC clone J_AA005I07 |
| 52 | 98.927 | 1118 | AC211865 | Gallus gallus BAC clone CH261-138N19 |
| 53 | 86.333 | 739 | AB556462 | Gallus gallus DNA, CENP-A associated sequence |
| 54 | 99.176 | 1092 | AC188809 | Gallus gallus BAC clone CH261-16A5 |
| 55 | 95.871 | 557 | AC270405 | Gallus gallus fosmid J_AD-488G17 |
| 56 | 92.722 | 632 | AB556456 | Gallus gallus DNA, CENP-A associated sequence |
| 57 | 98.262 | 633 | AB556456 | Gallus gallus DNA, CENP-A associated sequence |
| 58 | 99.45 | 909 | X57344 | G.gallus repetitive DNA |
| 59 | 91.009 | 634 | X57344 | G.gallus repetitive DNA |
| 60 | 93.046 | 302 | AC270447 | Gallus gallus fosmid J_AD-49G16 |
| 61 | 93.023 | 301 | AC270447 | Gallus gallus fosmid J_AD-49G16 |
| 62 | 92.515 | 334 | AM982513 | Gallus gallus partial genomic XhoI repeat element |
| 63 | 99.554 | 224 | X57344 | G.gallus repetitive DNA |

^a^Accession number of the best match genome

^b^Name of the best match genome
