# Supplementary material for: Whole-genome based strain identification of fowlpox virus directly from cutaneous tissue and propagated virus
Source: PLoS One. 2021 Dec 16;16(12):e0261122. doi: 10.1371/journal.pone.0261122 (PMC8675702; doi:10.1371/journal.pone.0261122)
Supplement: S3 Table — (DOCX) [file pone.0261122.s003.docx]

**S3 Table. Analysis of FPV-CAMs Nanopore *de novo* assembly contigs with BLAST**.

| Contigs | % Identity | Alignment length | Subject Accession^a^ | Subject title^b^ |
| --- | --- | --- | --- | --- |
| 1 | 99.02 | 49162 | AF198100 | Fowlpox virus, complete genome |
| 2 | 98.709 | 14325 | MH709125 | Fowlpox virus isolate FWPV-MN00.1, complete genome |
| 3 | 98.812 | 17086 | AF198100 | Fowlpox virus, complete genome |
| 4 | 98.872 | 40783 | MH709124 | Fowlpox virus isolate FWPV-MN00.2, complete genome |
| 5 | 98.822 | 20368 | AF198100 | Fowlpox virus, complete genome |
| 6 | 98.607 | 13350 | MH879470 | Gallus gallus mitochondrion, complete genome |
| 7 | 98.96 | 51622 | AF198100 | Fowlpox virus, complete genome |
| 8 | 99.13 | 12869 | AF198100 | Fowlpox virus, complete genome |

^a^Accession number of the best match genome

^b^Name of the best match genome
