# Supplementary material for: Whole-genome based strain identification of fowlpox virus directly from cutaneous tissue and propagated virus
Source: PLoS One. 2021 Dec 16;16(12):e0261122. doi: 10.1371/journal.pone.0261122 (PMC8675702; doi:10.1371/journal.pone.0261122)
Supplement: S4 Table — (DOCX) [file pone.0261122.s004.docx]

**S4 Table. Analysis of FPV-COMB hybrid assembly contigs with BLAST.**

| Contigs | % Identity | Alignment length | Subject Accession^a^ | Subject title^b^ |
| --- | --- | --- | --- | --- |
| 1 | 99.867 | 86169 | MH709124 | Fowlpox virus isolate FWPV-MN00.2, complete genome |
| 2 | 99.947 | 78074 | AF198100 | Fowlpox virus, complete genome |
| 3 | 99.956 | 50282 | AF198100 | Fowlpox virus, complete genome |
| 4 | 100 | 7834 | AJ581527 | Fowlpox virus (isolate HP-438[Munich]), passage 438 clone FP9, complete genome |
| 5 | 97.002 | 5303 | AC231413 | Gallus gallus BAC clone CH261-89E17 |
| 6 | 100 | 785 | BX934248 | Gallus gallus finished cDNA |
| 7 | 97.383 | 5158 | AC192626 | Gallus gallus BAC clone CH261-8I22 |
| 8 | 97.006 | 1670 | XR_003073022 | Gallus gallus uncharacterized |
| 9 | 98.103 | 3954 | AC275676 | gallus gallus BAC clone J_AA005I07 |
| 10 | 99.791 | 1914 | XM_025145577 | Gallus gallus serine/arginine repetitive matrix protein |
| 11 | 99.031 | 1032 | XM_025146541 | Gallus gallus coiled-coil domain-containing protein |
| 12 | 90.472 | 1123 | XM_015290599 | Gallus gallus uncharacterized |
| 13 | 84.689 | 1143 | XM_021381768 | Numida meleagris coiled-coil domain-containing protein |
| 14 | 99.552 | 1340 | AB556728 | Gallus gallus DNA |
| 15 | 86.33 | 1368 | AC239805 | Gallus gallus BAC clone CH261-60P24 |
| 16 | 99.496 | 595 | AB556513 | Gallus gallus DNA, CENP-A associated sequence |
| 17 | 98.726 | 1491 | AB556723 | Gallus gallus DNA |
| 18 | 97.422 | 2521 | AC232992 | Gallus gallus FOSMID clone J_AD-669E23 |
| 19 | 98.806 | 2428 | AC275676 | gallus gallus BAC clone J_AA005I07 |
| 20 | 98.446 | 1094 | XM_015302003 | PREDICTED: Gallus gallus zinc finger CCCH domain-containing protein |
| 21 | 97.293 | 1182 | X57344 | G.gallus repetitive DNA |
| 22 | 88.509 | 1462 | XM_025145357 | Gallus gallus olfactory receptor 14A16-like 33 |
| 23 | 96.881 | 2244 | AC212253 | Gallus gallus BAC clone CH261-163C7 |
| 24 | 83.907 | 1684 | AC186824 | Gallus gallus BAC clone CH261-143N19 |
| 25 | 90.088 | 797 | XM_025145979 | Gallus gallus zinc finger protein |
| 26 | 87.831 | 756 | AB556728 | Gallus gallus DNA |
| 27 | 97.966 | 2016 | AC200646 | Gallus gallus BAC clone CH261-126P15 |
| 28 | 97.906 | 1910 | AC215792 | Gallus gallus BAC clone CH261-98P16 from chromosome z, complete sequence |
| 29 | 98.49 | 596 | XR_003071684 | Gallus gallus uncharacterized |
| 30 | 97.332 | 1874 | CP009623 | Staphylococcus agnetis strain 908, complete genome |
| 31 | 97.222 | 648 | XM_025146102 | Gallus gallus coiled-coil domain-containing protein |
| 32 | 99.775 | 1774 | AB556734 | Gallus gallus DNA |
| 33 | 95.3 | 1085 | XM_015273962 | Gallus gallus SUN domain-containing protein |
| 34 | 96.579 | 1754 | GU261698 | Gallus gallus isolate yin21 breed Autochthonic chicken mitochondrion |
| 35 | 99.665 | 1195 | MG967540 | Gallus gallus 5' external transcribed spacer 18S ribosomal RNA gene |
| 36 | 99.243 | 1717 | AC275676 | gallus gallus BAC clone J_AA005I07 |
| 37 | 97.672 | 1718 | AC216221 | Gallus gallus BAC clone CH261-8M6 |
| 38 | 99.941 | 1691 | CP009623 | Staphylococcus agnetis strain 908, complete genome |
| 39 | 100 | 986 | AB556726 | Gallus gallus DNA |
| 40 | 99.445 | 1621 | XR_003075701 | Gallus gallus uncharacterized |
| 41 | 100 | 1605 | CP009623 | Staphylococcus agnetis strain 908, complete genome |
| 42 | 84.269 | 998 | X57344 | G.gallus repetitive DNA |
| 43 | 99.868 | 1519 | CP009623 | Staphylococcus agnetis strain 908, complete genome |
| 44 | 96.701 | 1455 | AC275646 | gallus gallus BAC clone J_AA133E17 from chromosome unknown |
| 45 | 99.384 | 487 | XR_003072638 | Gallus gallus olfactory receptor 14J1-like 2 |
| 46 | 98.696 | 230 | XR_003075861 | Gallus gallus uncharacterized |
| 47 | 93.615 | 877 | AC275676 | gallus gallus BAC clone J_AA005I07 |
| 48 | 99.37 | 476 | XM_025148008 | Gallus gallus SUN domain-containing protein |
| 49 | 100 | 1378 | XR_003078040 | Gallus gallus 28S ribosomal RNA |
| 50 | 91.701 | 735 | XR_003072651 | Gallus gallus uncharacterized LOC107049478 |
| 51 | 100 | 1356 | CP009624 | Staphylococcus agnetis strain 908 plasmid unamed, complete sequence |
| 52 | 99.852 | 1354 | CP009623 | Staphylococcus agnetis strain 908, complete genome |
| 53 | 99.263 | 1356 | CP009623 | Staphylococcus agnetis strain 908, complete genome |
| 54 | 100 | 1303 | CP009623 | Staphylococcus agnetis strain 908, complete genome |
| 55 | 83.824 | 204 | AC189679 | Gallus gallus BAC clone CH261-81E3 from chromosome z, complete sequence |
| 56 | 88.075 | 696 | XR_003073180 | Gallus gallus uncharacterized |
| 57 | 100 | 1217 | CP009623 | Staphylococcus agnetis strain 908, complete genome |
| 58 | 97.697 | 1216 | AC211865 | Gallus gallus BAC clone CH261-138N19 |
| 59 | 99.585 | 1205 | CP009623 | Staphylococcus agnetis strain 908, complete genome |
| 60 | 75.519 | 482 | AF401308 | Gallus gallus clone pG193_T3-end CNM repeat sequence |
| 61 | 86.957 | 943 | X57344 | G.gallus repetitive DNA |
| 62 | 100 | 1149 | CP009623 | Staphylococcus agnetis strain 908, complete genome |
| 63 | 89.481 | 770 | XR_001468564 | Gallus gallus PHD finger protein |
| 64 | 100 | 1118 | CP009623 | Staphylococcus agnetis strain 908, complete genome |
| 65 | 99.723 | 1083 | AC270444 | Gallus gallus fosmid J_AE-110L17 |
| 66 | 100 | 1059 | CP009624 | Staphylococcus agnetis strain 908 plasmid unamed, complete sequence |
| 67 | 97.421 | 1047 | AC275676 | gallus gallus BAC clone J_AA005I07 from chromosome unknown, complete sequence |
| 68 | 100 | 1040 | CP009623 | Staphylococcus agnetis strain 908, complete genome |
| 69 | 100 | 126 | XM_025144072 | Gallus gallus uncharacterized |
| 70 | 99.805 | 1024 | CP009623 | Staphylococcus agnetis strain 908, complete genome |
| 71 | 99.901 | 1015 | KT445934 | Gallus gallus 5' external transcribed spacer, 18S ribosomal RNA gene |
| 72 | 99.276 | 691 | CP009624 | Staphylococcus agnetis strain 908 plasmid unamed, complete sequence |
| 73 | 99.901 | 1006 | AC234792 | Gallus gallus FOSMID clone J_AD-450B22 |
| 74 | 98.276 | 986 | AY489052 | Gallus gallus clone PG6420 satellite sequence |
| 75 | 93.4 | 803 | MG967540 | Gallus gallus 5' external transcribed spacer 18S ribosomal RNA gene |
| 76 | 98.745 | 239 | XR_003072881 | Gallus gallus uncharacterized |
| 77 | 98.271 | 347 | AF124927 | Gallus gallus clone pG6416 inverted repeat region |

^a^Accession number of the best match genome

^b^Name of the best match genome
