# Supplementary material for: Whole-genome based strain identification of fowlpox virus directly from cutaneous tissue and propagated virus
Source: PLoS One. 2021 Dec 16;16(12):e0261122. doi: 10.1371/journal.pone.0261122 (PMC8675702; doi:10.1371/journal.pone.0261122)
Supplement: S5 Table — (DOCX) [file pone.0261122.s005.docx]

**S5 Table. Analysis of FPV-COMB Illumina *de novo* assembly contigs with BLAST.**

| Contigs | % Identity | Alignment length | Subject Accession^a^ | Subject title^b^ |
| --- | --- | --- | --- | --- |
| 1 | 99.919 | 98371 | MH709124 | Fowlpox virus isolate FWPV-MN00.2, complete genome |
| 2 | 99.947 | 70337 | AF198100 | Fowlpox virus, complete genome |
| 3 | 99.956 | 50282 | AF198100 | Fowlpox virus, complete genome |
| 4 | 97.271 | 9565 | AC189661 | Gallus gallus BAC clone CH261-113D21 |
| 5 | 97.169 | 8335 | AC205869 | Gallus gallus BAC clone CH261-9B2 |
| 6 | 100 | 7834 | AJ581527 | Fowlpox virus (isolate HP-438[Munich]), passage 438 clone FP9 |
| 7 | 99.283 | 6419 | CP009623 | Staphylococcus agnetis strain 908 |
| 8 | 100 | 785 | BX934248 | Gallus gallus finished cDNA |
| 9 | 96.116 | 5072 | AC213406 | Gallus gallus BAC clone TAM32-24P1 |
| 10 | 98.179 | 5930 | AC270364 | Gallus gallus fosmid J_AD-67N6 |
| 11 | 98.3 | 5648 | AC214807 | Gallus gallus BAC clone CH261-99G18 |
| 12 | 99.34 | 3486 | AC270329 | Gallus gallus fosmid J_AE-33C7 |
| 13 | 93.461 | 3563 | AC213026 | Gallus gallus BAC clone CH261-122C6 |
| 14 | 97.688 | 4368 | AC275676 | gallus gallus BAC clone J_AA005I07 |
| 15 | 97.112 | 3913 | AC275676 | gallus gallus BAC clone J_AA005I07 |
| 16 | 99.031 | 1032 | XM_025146541 | Gallus gallus coiled-coil domain-containing protein |
| 17 | 98.723 | 3368 | AC270341 | Gallus gallus fosmid J_AD-221E18 |
| 18 | 97.375 | 1981 | XM_015302003 | Gallus gallus zinc finger CCCH domain-containing protein |
| 19 | 90.472 | 1123 | XM_015290599 | Gallus gallus uncharacterized |
| 20 | 98.808 | 3103 | AC233985 | Gallus gallus BAC clone CH261-168N7 |
| 21 | 84.689 | 1143 | XM_021381768 | Numida meleagris coiled-coil domain-containing protein |
| 22 | 98.992 | 2083 | AB556723 | Gallus gallus DNA, chromosome 2, |
| 23 | 99.496 | 595 | AB556513 | Gallus gallus DNA, CENP-A associated sequence |
| 24 | 97.666 | 2399 | AC214807 | Gallus gallus BAC clone CH261-99G18 |
| 25 | 99.665 | 1195 | MG967540 | Gallus gallus 5' external transcribed spacer 18S ribosomal RNA gene |
| 26 | 94.592 | 980 | XR_003073198 | Gallus gallus uncharacterized |
| 27 | 93.675 | 1834 | AC201947 | Gallus gallus BAC clone CH261-7E21 |
| 28 | 99.96 | 2505 | AC232992 | Gallus gallus FOSMID clone J_AD-669E23 |
| 29 | 98.806 | 2428 | AC275676 | gallus gallus BAC clone J_AA005I07 |
| 30 | 99.05 | 2316 | AC275646 | gallus gallus BAC clone J_AA133E17 |
| 31 | 98.65 | 2297 | AC275676 | gallus gallus BAC clone J_AA005I07 |
| 32 | 88.509 | 1462 | XM_025145357 | Gallus gallus olfactory receptor 14A16-like 33 |
| 33 | 99.166 | 1918 | XM_025145577 | Gallus gallus serine/arginine repetitive matrix protein 1-like (LOC112530937) |
| 34 | 83.824 | 204 | AC189679 | Gallus gallus BAC clone CH261-81E3 |
| 35 | 98.276 | 1566 | XM_025144243 | Gallus gallus inositol 1,4,5-trisphosphate receptor-interacting protein-like 1 |
| 36 | 90.088 | 797 | XM_025145979 | Gallus gallus zinc finger protein 624 (ZNF624) |
| 37 | 99.57 | 2093 | AC214807 | Gallus gallus BAC clone CH261-99G18 |
| 38 | 97.966 | 2016 | AC200646 | Gallus gallus BAC clone CH261-126P15 |
| 39 | 99.791 | 1438 | AB556724 | Gallus gallus DNA |
| 40 | 98.696 | 230 | XR_003075861 | Gallus gallus uncharacterized |
| 41 | 99.78 | 1820 | AB556726 | Gallus gallus DNA |
| 42 | 98.49 | 596 | XR_003071684 | Gallus gallus uncharacterized |
| 43 | 86.31 | 1366 | AC239805 | Gallus gallus BAC clone CH261-60P24 |
| 44 | 98.472 | 1832 | AC186352 | Gallus gallus BAC clone CH261-143E22 |
| 45 | 85.99 | 621 | AB556456 | Gallus gallus DNA, CENP-A associated sequence |
| 46 | 99.887 | 1772 | AB556734 | Gallus gallus DNA |
| 47 | 95.575 | 791 | AC270371 | Gallus gallus fosmid J_AD-837K11 |
| 48 | 95.3 | 1085 | XM_015273962 | Gallus gallus SUN domain- |
| 49 | 97.266 | 512 | XM_025146102 | Gallus gallus coiled-coil domain-containing protein 81-like (LOC107049387) |
| 50 | 88.732 | 71 | AC270447 | Gallus gallus fosmid J_AD-49G16 |
| 51 | 99.941 | 1691 | CP009623 | Staphylococcus agnetis strain 908 |
| 52 | 90.083 | 1331 | AC186851 | Gallus gallus BAC clone CH261-18A24 |
| 53 | 95.235 | 1406 | XR_003073179 | Gallus gallus uncharacterized |
| 54 | 99.445 | 1621 | XR_003075701 | Gallus gallus uncharacterized LOC112532620 |
| 55 | 97.271 | 916 | XM_025150486 | Gallus gallus uncharacterized |
| 56 | 99.808 | 521 | XM_025145552 | Gallus gallus serine/arginine repetitive matrix protein |
| 57 | 100 | 1605 | CP009623 | Staphylococcus agnetis strain 908, complete genome |
| 58 | 100 | 1603 | CP009623 | Staphylococcus agnetis strain 908, complete genome |
| 59 | 88.889 | 1413 | AC233986 | Gallus gallus BAC clone CH261-185L12 |
| 60 | 99.773 | 1323 | AB556728 | Gallus gallus DNA |
| 61 | 96.067 | 1500 | MG967540 | Gallus gallus 5' external transcribed spacer 18S ribosomal RNA gene |
| 62 | 89.206 | 630 | X57344 | G.gallus repetitive DNA |
| 63 | 99.868 | 1519 | CP009623 | Staphylococcus agnetis strain 908, complete genome |
| 64 | 100 | 908 | XR_003073353 | Gallus gallus uncharacterized |
| 65 | 98.05 | 923 | XR_003073197 | Gallus gallus uncharacterized |
| 66 | 99.793 | 1452 | AB556728 | Gallus gallus DNA |
| 67 | 99.384 | 487 | XR_003072638 | Gallus gallus olfactory receptor 14J1-like 2 (OR14J1L2) |
| 68 | 98.167 | 1200 | XR_003072022 | Gallus gallus uncharacterized |
| 69 | 98.183 | 1431 | AC188439 | Gallus gallus BAC clone CH261-21D19 |
| 70 | 99.526 | 633 | XR_003073352 | Gallus gallus uncharacterized |
| 71 | 100 | 1378 | XR_003078040 | Gallus gallus 28S ribosomal |
| 72 | 97.89 | 616 | XM_025146561 | Gallus gallus guanylate-binding protein 2-like |
| 73 | 99.273 | 1376 | AC270379 | Gallus gallus fosmid J_AD-672N11 |
| 74 | 76.16 | 625 | AB556456 | Gallus gallus DNA, CENP-A associated sequence |
| 75 | 96.225 | 1351 | AC244202 | Gallus gallus BAC clone TAM32-26I15 |
| 76 | 100 | 1356 | CP009624 | Staphylococcus agnetis strain 908 plasmid |
| 77 | 99.852 | 1354 | CP009623 | Staphylococcus agnetis strain 908, complete genome |
| 78 | 99.263 | 1356 | CP009623 | Staphylococcus agnetis strain 908, complete genome |
| 79 | 99.017 | 1323 | AC275646 | gallus gallus BAC clone J_AA133E17 from chromosome unknown, complete sequence |
| 80 | 100 | 1303 | CP009623 | Staphylococcus agnetis strain 908, complete genome |
| 81 | 100 | 1275 | XR_003078040 | Gallus gallus 28S ribosomal RNA |
| 82 | 98.526 | 814 | XR_003073208 | Gallus gallus uncharacterized |
| 83 | 87.469 | 798 | XR_003073180 | Gallus gallus uncharacterized |
| 84 | 99.918 | 1220 | KT445934 | Gallus gallus 5' external transcribed spacer, 18S ribosomal RNA gene |
| 85 | 100 | 1217 | CP009623 | Staphylococcus agnetis strain 908, complete genome |
| 86 | 97.697 | 1216 | AC211865 | Gallus gallus BAC clone CH261-138N19 |
| 87 | 99.2 | 750 | X57344 | G.gallus repetitive DNA |
| 88 | 99.585 | 1205 | CP009623 | Staphylococcus agnetis strain 908, complete genome |
| 89 | 100 | 187 | XM_025145667 | Gallus gallus serine/arginine repetitive matrix protein |
| 90 | 97.196 | 1177 | AC243549 | Gallus gallus BAC clone CH261-69N2 |
| 91 | 87.063 | 943 | X57344 | G.gallus repetitive DNA |
| 92 | 95.559 | 1171 | AC275646 | gallus gallus BAC clone J_AA133E17 |
| 93 | 100 | 1149 | CP009623 | Staphylococcus agnetis strain 908, complete genome |
| 94 | 98.229 | 621 | AB556668 | Gallus gallus DNA, CENP-A associated sequence |
| 95 | 88.221 | 832 | AC270447 | Gallus gallus fosmid J_AD-49G16, complete sequence |
| 96 | 99.911 | 1124 | CP009623 | Staphylococcus agnetis strain 908, complete genome |
| 97 | 100 | 1118 | CP009623 | Staphylococcus agnetis strain 908, complete genome |
| 98 | 98.995 | 1094 | AC275676 | gallus gallus BAC clone J_AA005I07 from chromosome unknown, complete sequence |
| 99 | 87.831 | 756 | AB556728 | Gallus gallus DNA, chromosome 11, centromere 11 repeat sequence |
| 100 | 99.723 | 1083 | AC270444 | Gallus gallus fosmid J_AE-110L17, complete sequence |
| 101 | 98.481 | 395 | AB556531 | Gallus gallus DNA, CENP-A associated sequence |
| 102 | 97.763 | 447 | AB556671 | Gallus gallus DNA, CENP-A associated sequence |
| 103 | 93.997 | 633 | AB556456 | Gallus gallus DNA, CENP-A associated sequence |
| 104 | 99.907 | 1073 | AC234792 | Gallus gallus FOSMID clone J_AD-450B22 |
| 105 | 100 | 1060 | KT445934 | Gallus gallus 5' external transcribed spacer, 18S ribosomal RNA gene |
| 106 | 100 | 1059 | CP009624 | Staphylococcus agnetis strain 908 plasmid unamed, complete sequence |
| 107 | 100 | 1040 | CP009623 | Staphylococcus agnetis strain 908, complete genome |
| 108 | 100 | 126 | XM_025144072 | Gallus gallus uncharacterized |
| 109 | 97.649 | 1021 | AC275646 | gallus gallus BAC clone J_AA133E17 |
| 110 | 99.805 | 1026 | XR_001464683 | Gallus gallus uncharacterized |
| 111 | 99.805 | 1024 | CP009623 | Staphylococcus agnetis strain 908, complete genome |
| 112 | 89.286 | 364 | AC163710 | Gallus gallus BAC clone TAM31-16O5 |
| 113 | 99.276 | 691 | CP009624 | Staphylococcus agnetis strain 908 plasmid |
| 114 | 98.377 | 986 | AY489052 | Gallus gallus clone PG6420 satellite sequence |
| 115 | 93.023 | 301 | AC270447 | Gallus gallus fosmid J_AD-49G16 |
| 116 | 93.046 | 302 | AC270447 | Gallus gallus fosmid J_AD-49G16, complete sequence |
| 117 | 98.446 | 386 | AF124927 | Gallus gallus clone pG6416 inverted repeat region |
| 118 | 92.857 | 350 | M24755 | Gallus gallus clone pUGD0601 W |

^a^Accession number of the best match genome

^b^Name of the best match genome
