# Supplementary material for: Whole-genome based strain identification of fowlpox virus directly from cutaneous tissue and propagated virus
Source: PLoS One. 2021 Dec 16;16(12):e0261122. doi: 10.1371/journal.pone.0261122 (PMC8675702; doi:10.1371/journal.pone.0261122)
Supplement: S6 Table — (DOCX) [file pone.0261122.s006.docx]

**S6 Table. Analysis of FPV-COMB Nanopore *de novo* assembly contigs with BLAST.**

| Contigs | % Identity | Alignment length | Subject Accession^a^ | Subject title^b^ |
| --- | --- | --- | --- | --- |
| 1 | 97.714 | 12863 | KU310942 | Fowl aviadenovirus D isolate ON P2, complete genome |
| 2 | 96.755 | 493 | AB556668 | Gallus gallus DNA, CENP-A associated sequence, partial sequence, clone: CAIP#397 |
| 3 | 90.063 | 634 | AB556456 | Gallus gallus DNA, CENP-A associated sequence, partial sequence, clone: CAIP#152 |

^a^Accession number of the best match genome

^b^Name of the best match genome
